# Open Porosity and Pore Size Distribution of Mesoporous Silica Films Investigated by Positron Annihilation Lifetime Spectroscopy and Ellipsometric Porosimetry

**DOI:** 10.3390/ma14123371

**Published:** 2021-06-18

**Authors:** Bangyun Xiong, Jingjing Li, Chunqing He, Jiale Lai, Xiangjia Liu, Tao Huang

**Affiliations:** 1 Guangdong Key Laboratory for Hydrogen Energy Technologies, School of Materials Science and Hydrogen Energy, Foshan University, Foshan 528000, China; lijingjing0842@163.com (J.L.); sg1010594845@163.com (J.L.); liuxiangjia6@163.com (X.L.); huangtao9902@163.com (T.H.); 2 Key Laboratory of Nuclear Solid State Physics Hubei Province, School of Physics and Technology, Wuhan University, Wuhan 430072, China

**Keywords:** open porosity, mesopore size, orthopositronium lifetime, positron annihilation lifetime spectroscopy, ellipsometric porosimetry

## Abstract

Tunable mesoporous silica films were prepared though a sol-gel process directed by the self-assembly of various triblock copolymers. Positron annihilation γ-ray energy spectroscopy and positron annihilation lifetime spectroscopy (PALS) based on intense pulsed slow positron beams as well as ellipsometric porosimetry (EP) combined with heptane adsorption were utilized to characterize the open porosity/interconnectivity and pore size distribution for the prepared films. The consistency between the open porosities was examined by the variations of orthopositronium (o-Ps) 3γ annihilation fractions and the total adsorbed volumes of heptane. The average pore sizes deduced by PALS from the longest-lived o-Ps lifetimes are in good agreement with those by EP on the basis of the Barrett–Joyner–Halenda model, as indicated by a well fitted line of slope *k* = 1. The results indicate that the EP combined with heptane adsorption is a useful method with high sensitivity for calibrating the mesopore size in highly interconnected mesoporous films, whereas PALS is a novel, complementary tool for characterizing both closed and open pores in them.

## 1. Introduction

Mesoporous silica films have been extensively developed as separations, low-dielectric interlayers, catalysts, gas sensors and adsorbents. Accurate control of the pore structure of mesoporous silica is one of the most important issues for realizing its practical application or expanding its specific application scope. The introduction of nanoscaled pores into silica films has aroused great interest by using nonionic triblock copolymers [1,2,3,4] as structural templates to tailor the pore size and morphology, benefitting from the self-organizing ability of amphiphilic copolymers [4]. Meanwhile, accurate characterization of pore structures is of great significance to improve the performance of functional silica films with specific pore characteristics.

However, it is difficult for most traditional techniques to characterize nanopores in submicron thin films fabricated on thick solid substrates. For instance, transmission electron microscopy (TEM) is a common resort to probe the pore morphology of films [5]. However, the films must be scraped or sliced from the substrates, resulting in fragmentation, which may not reflect the porosity of the intact films. Positron annihilation γ-ray energy spectroscopy (PAES) [6,7] and positron annihilation lifetime spectroscopy (PALS) [8,9,10] based on an intense pulsed positron beam have been proven as powerful and sensitive tools to elucidate the nanoporosity of thin films on substrates. Ellipsometric porosimetry (EP) combined with vapor adsorption has been successfully used to evaluate the open porosity of thin films [5,11]. Therefore, the combined application of PAES, PALS and EP is expected to provide more detailed and reliable characterization for the difference in the mesostructures of silica films.

In the present work, controllable mesoporous silica films were fabricated with the assistance of different BASF triblock copolymers via a sol-gel route. The pore structures of the fabricated films, such as pore interconnectivity/open porosity and pore size, were examined by means of PAES, PALS and EP. The feasibility of PALS based on a slow positron beam for accurately characterizing both closed and open pores in thin silica films was verified by comparative studies on their mesoporosities measured by both PALS and EP combined with vapor adsorption.

## 2. Materials and Methods

### 2.1. Preparation of Mesoporous Silica Films

A sol-gel process in accordance with the reported procedure [12] was applied to synthesize mesoporous silica films with adjustable mesopore sizes and morphologies. Tetraethoxysilane (TEOS) was selected as the silica skeleton precursor. Amphiphilic BASF Pluronic surfactants such as triblock copolymers F127 (EO_106_PO_70_EO_106_, M_w_ = 12,600 g/mol), F88 (EO_100_PO_39_EO_100_, M_w_ = 11,400 g/mol), F38 (EO_43_PO_14_EO_43_, M_w_ = 4700 g/mol) and P103 (EO_17_PO_85_EO_17_, M_w_ = 4950 g/mol) were introduced as the templating agents, respectively. A surfactant solution was prepared by dissolving a fixed amount of 3.02 g BASF surfactant (denoted by EO*_x_*PO*_y_*EO*_x_*, where *x* and *y* are determined by the above triblock copolymers) in 40 mL anhydrous alcohol (EtOH). Typically, a precursor solution containing TEOS, EtOH, HCl and H_2_O was developed under stirring at 100 °C. The precursor solution was aged at 100 °C for 30 min followed by the addition of the above surfactant solution. The mixed solution was continuously stirred for 1 h. The masses of the respective compositions in the final sols were 3.02 g EO*_x_*PO*_y_*EO*_x_*, 8.33 g TEOS, 55.28 g EtOH, 5.76 g H_2_O, and 0.0042 g HCl. The mass ratio of EO*_x_*PO*_y_*EO*_x_* to TEOS was around 36.25%. The weight ratio of EO*_x_*PO*_y_*EO*_x_* in all final sols was calculated to be about 4.2 wt%.

The final precursor sol was subsequently deposited on a polished monocrystalline silicon (100) wafer by dip-coating [13,14] with a speed of 30 cm/min. The as-deposited film was then cured at 100 °C for 3 h and finally calcined at 450 °C for 3 h to decompose the surfactant template. For convenience, the mesoporous silica thin films were represented by their corresponding surfactant names. Further, an additional set of the same films were capped by nonporous SiO_2_ layers (about 20 nm in thickness) through electron-beam sputtering [15] for PALS experiments.

### 2.2. PAES and PALS Based on Intense Pulsed Slow Positron Beams

All pore characterization experiments were done at the National Institute of Advanced Industrial Science and Technology, Tsukuba, Japan. Positron annihilation γ-ray energy spectra were conducted by a variable-energy pulsed slow positron beam with a Ge detector [5,7], both for the uncapped silica films and capped ones. The orthopositronium (o-Ps) 3γ annihilation fraction (*I*_3γ_) was estimated from the recorded γ-ray energy spectrum as described elsewhere [16,17,18,19]. Positron annihilation lifetime spectra of the capped silica films were collected using an intense pulsed positron beam [11,12,15,20]. The incident positron energy (*E*_in_) was fixed at 2 keV, at which the vast majority of positrons were injected into the inside thin films. The total annihilation event counts for each spectrum was approximately 5 million. The time resolution was around 330 ps. Background subtraction was performed by using the positron annihilation lifetime spectrum of Kapton film for reference [3]. All films were heated at 250 °C for 30 min in nitrogen (N_2_) atmosphere to eliminate some adsorbed water before the positron annihilation measurements. A continuous o-Ps lifetime distribution was deduced from the measured positron annihilation lifetime spectra by CONTIN program [21]. On the basis of the rectangular Tao-Eldrup (RTE) model [10,22,23], the mesopore side length (*a*_PALS_) was computed from the longest-lived lifetime of o-Ps.

### 2.3. Ellipsometric Porosimetry Combined with Heptane Adsorption

The schematic diagram of the apparatus of heptane adsorption is available in the supplementary materials. A silica thin film was installed in the sample chamber equipped with an ellipsometric porosimeter, an ultrahigh vacuum pump, a temperature control console and a system for dosing heptane. The sample chamber was degassed to 10^−5^ torr, and the film was heated at 250 °C for 30 min. When the temperature dropped to 30 °C, heptane was introduced into the chamber up to a certain pressure (*p*), which is in the range of 0.02~46.88 torr. The partial pressure (*p*/*p*_0_) is defined as the ratio of the heptane pressure (*p*) to the saturated vapor pressure of heptane (*p*_0_), ranging from 0.003 to 0.803 determined by the dosing heptane. For each *p*/*p*_0_, the ellipsometric spectrum was measured at 30 °C for the film filled by heptane. The characteristic parameters Ψ and ∆ were fitted over wavelengths from 300 to 800 nm assuming the Cauchy model [24]. The observed refractive index (*n*_o_) at 630 nm was computed from the fitted ellipsometric parameters upon successive heptane adsorption-desorption versus *p*/*p*_0_. The pore volume fractions filled with heptane (*V*_filled_) were estimated in accordance with the following deduced Lorentz–Lorenz formula [25]:

(1) $$V_{\mathrm{filled}}=\left(\frac{n_{o}^{2}-1}{n_{o}^{2}+2}-\frac{n_{f}^{2}-1}{n_{f}^{2}+2}\right)/\left(\frac{n_{a}^{2}-1}{n_{a}^{2}+2}\right)$$

In this study, *n_f_* refers to the fixed refractive index of the silica film at *p*/*p*_0_ = 0 and *n_a_* represents the refractive index of adsorbate (*n*_heptane_ = 1.386) [25,26]. Hence, the physical adsorption isotherms of heptane were measured by the changes of *V*_filled_ values with *p*/*p*_0_ according to Equation (1) with the measured *n*_o_. The open porosities of the films were associated with the total adsorbed volumes of heptane at the high *p*/*p*_0_ regions.

Starting from the vacuum, as the *p*/*p*_0_ increases, the micropores (pore radius below 1 nm) at first are filled by the heptane. The pore surfaces are occupied layer-by-layer by adsorption. The thickness of the adsorbed layer (*t*) increases progressively with *p*/*p*_0_, which can be expressed as [25]

(2) $$t=5t_{m}\mathrm{lg}\frac{p}{p_{0}}$$

where *t_m_* denotes the thickness of monolayer adsorption of heptane, and 0.38 nm was applied to the value of *t_m_* in Equation (2) [27]. For higher *p*/*p*_0_, the capillary condensation takes place. The Kelvin radius (*r_K_*) is determined by the following Kelvin equation [28,29]:

(3) $$r_{K}=\frac{2\gamma V_{L}}{RT}\ln\frac{p}{p_{0}}$$

where *γ* and *V_L_* refer to the surface tension and molar volume of heptane, respectively. *R* stands for the ideal gas constant. *T* is the temperature, *T* = 303 K for the present work. Capillary condensation happens in mesopores. Under the assumption of the cylindrical pores, the pore radius (*R*) was therefore estimated by the *r_K_* and *t*,

(4) $$R=r_{K}+t$$

From the physisorption isotherms, the *V*_filled_ is a function of *p*/*p*_0_, namely *V*_filled_ = *f* (*p*/*p*_0_). Thus, *R* = *f* (*p*/*p*_0_) is evaluated. The pore size distribution (PSD) is calculated as the change of d*V*/d*R* with *R* from the desorption data. To the first order approximation, under assumption of a symmetrical and unimodal PSD, the average pore radius correlates with the maximum value of the PSD [29].

## 3. Results and Discussion

### 3.1. Pore Interconnectivity/Open Porosity of the Mesoporous Silica Films

**Figure 1:**
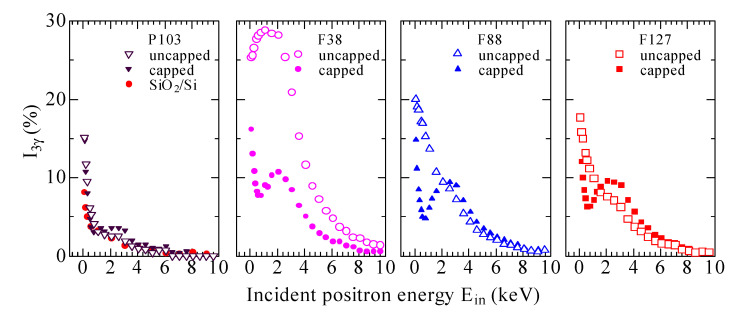
The *I*_3γ_-*E*_in_ curves both for the uncapped films prepared with various triblock copolymers (open symbols) and the corresponding capped ones (solid symbols). (The *I*_3γ_-*E*_in_ profiles for the films prepared with F38 and F127 are reprinted with permission from [5]. Copyright 2012 American Physical Society).

Figure 1 shows *I*_3γ_ as functions of *E*_in_ both for the uncapped films (open symbols) and capped ones (solid symbols), where the data for the F38 and F127 templated films were described in a previous paper [5]. As shown in a typical *I*_3γ_-*E*_in_ profile, such as for the uncapped F38 templated film, *I*_3γ_ climbs gradually as *E*_in_ increases to ~1.0 keV, due to o-Ps escaping from adjacent film surface and annihilation in vacuum. *I*_3γ_ almost remains unchanged with the increase of *E*_in_ up to ~2.5 keV, which is attributable to positronium (Ps) formation and Ps emission from the inside mesoporous film [7]. More and more positrons are injected into the silicon substrate with the further increase of *E*_in_. It is well known that there is no Ps formation in silicon so that *I*_3γ_ drops gradually to zero.

The uncapped films prepared with different templates show their own characteristics in *I*_3γ_ profiles (*E*_in_ < 3.0 keV). For the film with F38, the initial increase and gradual decrease of *I*_3γ_ suggests a high pore interconnectivity [7]. The initial increase and plateau of *I*_3γ_ disappear for the other three films. For the film prepared with P103, *I*_3γ_ falls sharply to a quite low value (~3%) with increasing *E*_in_ to ~1.0 keV, indicative of few Ps atoms capable of emitting out from the inside film with low pore interconnectivity [6]. High Ps diffusion coefficients through well-connected pores [7] are revealed by the gradual decline of *I*_3γ_ with the increase of *E*_in_ to ~3.0 keV for the films templated by F88 and F127. It is worth noting that for the two films, the open porosity/interconnectivity are very high, despite their lower *I*_3γ_ values in comparison with that for the film with F38. As was observed by the TEM images reported previously [5], worm-like pores and cage-like pores are formed in the film with F38 and F127, respectively. The larger cages are interconnected via smaller channels, which make thermalized Ps atoms likely confined in the larger cages and hardly able to pass through the smaller tubular channels between the cages to diffuse out from the films due to the Ps quantum confinement effect [5,30,31]. The results suggest that Ps diffusion is strongly affected both by open porosity/interconnectivity and pore morphology [32].

For the film prepared with P103, the *I*_3γ_-*E*_in_ curves are almost identical with and without capping a nonporous SiO_2_ layer on the film surface, which are likewise well coincided with the *I*_3γ_-*E*_in_ curve for the SiO_2_/Si film prepared without copolymer. The result directly evidences that most pores are closed in the film with P103. It is rational to observe the similar tendency of *I*_3γ_-*E*_in_ curves for the capped films with F38, F88 and F127 because the nonporous SiO_2_ capping layers inhibit o-Ps escaping from the films [5], except that the peak/plateau moves to higher *E*_in_ due to the increase of the film thickness. Therefore, the variations of *I*_3γ_ show that closed pores are fabricated in P103 templated film, but pores are well interconnected to the film surfaces in the other three ones.

**Figure 2:**
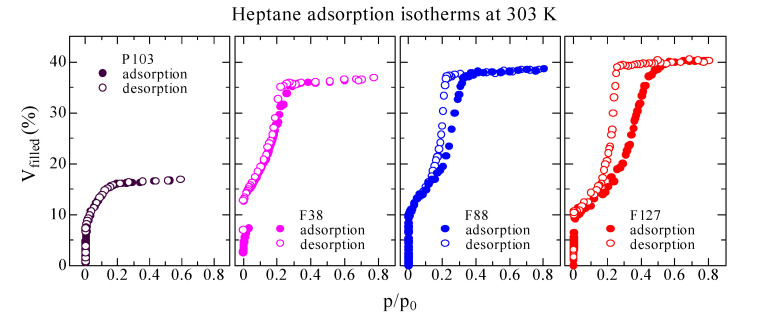
The heptane adsorption–desorption isotherms at 30 °C (303 K) of the mesoporous silica films prepared with P103, F38, F88 and F127, respectively. (The isotherms for the films with F38 and F127 are reprinted with permission from [5]. Copyright 2012 American Physical Society).

Figure 2 depicts the heptane adsorption–desorption isotherms at 303 K for the mesoporous films fabricated with different surfactants as functions of *p*/*p*_0_, where the ones templated by F38 and F127 were displayed previously [5]. The heptane physisorption isotherms of the silica films are found to be varied by the selection of different surfactants. For the three films except the film prepared with P103, the adsorption–desorption processes show a type IV isotherm, signifying mesostructures [33,34,35]. For the film templated by F38, a type pseudo-H1 hysteresis loop [36] is observed, likely indicative of disordered tube-like pores, confirmed with the worm-like pores by TEM [5]. The H1 loop exists in materials with narrow ranges of uniform mesopores [37]. However, type H5 hysteresis loops [37] are seen for the F88 and F127 templated films. The H5 loop exhibits a unique form related to special pore structures including both open and partly closed mesopores [37], consistent with the cage-like pores interconnected by the smaller tubular channels [5]. Moreover, the capillary condensation step at *p*/*p*_0_ moves from around 0.2 for the film template by F88 to about 0.3 for the one by F127, owing to the formation of larger mesopores in the latter.

Interestingly, for the film prepared with P103, *V*_filled_ increases sharply with increasing *p*/*p*_0_ from zero to about 0.2, and it keeps at a constant value with increasing *p*/*p*_0_ from ~0.2 to 0.6, corresponding to a reversible type I isotherm, a well-known Langmuir monolayer adsorption isotherm [38]. Type I isotherms exist in materials with a wider PSD, containing wider micropores and possibly narrower mesopores (of width < ~2.5 nm) [37]. The heptane gas is no longer adsorbed even though *p*/*p*_0_ is further increased to higher than 0.6, showing no larger open pores in the P103 templated film, as indicated by its absent hysteresis loop. *V*_filled_ reach constant values with increasing *p*/*p*_0_ above 0.2, 0.3, 0.35 and 0.5 for the film with P103, F38, F88 and F127, respectively, attributed to their open porosity/interconnectivity for corresponding silica films. The open porosities related to the total adsorbed volumes of heptane at the high *p*/*p*_0_ regions [11] are calculated to be 16.9, 37.0, 38.6 and 40.3%, for the films with P103, F38, F88 and F127, respectively. Among the four films, the highest open porosity/interconnectivity is revealed by EP in the film template by F127, coincident with the above discussion on the variation of *I*_3γ_.

### 3.2. Pore Size Distribution of the Mesoporous Silica Films

**Figure 3:**
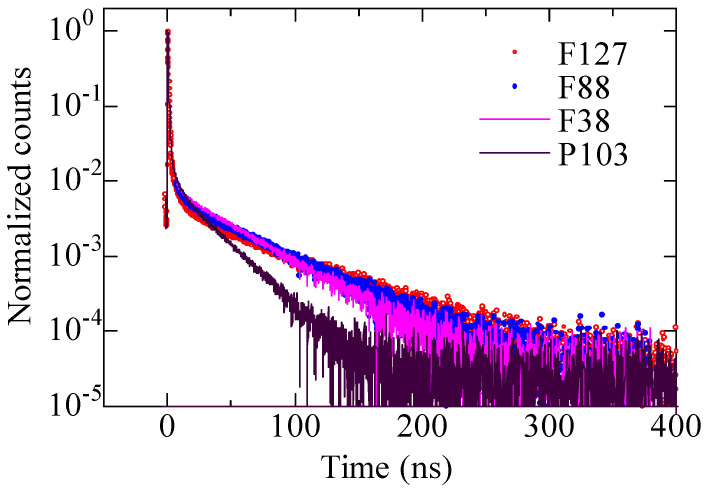
Positron annihilation lifetime spectra recorded at *E*_in_ = 2 keV for the capped mesoporous silica films prepared with P103, F38, F88 and F127, respectively. (Positron annihilation lifetime spectra for the films with P103, F38 and F127 are reprinted with permission from [12]. Copyright 2006 Elsevier).

Positron annihilation lifetime spectra recorded at *E*_in_ = 2 keV are demonstrated in Figure 3 for the capped films with different triblock copolymers, where those except for the F88 templated film were depicted previously [12]. Obviously, a longest-lived lifetime component resulted from o-Ps annihilation in mesopores can be seen for all films. The longest-lived components of the capped silica films change evidently, demonstrating adjustable pore sizes by the selection of templating agent [12]. The slope of o-Ps component for the films templated by various surfactants is in the order of F127 < F88 < F38 < P103, which shows that the average pore size is in the order of F127 > F88 > F38 > P103.

**Figure 4:**
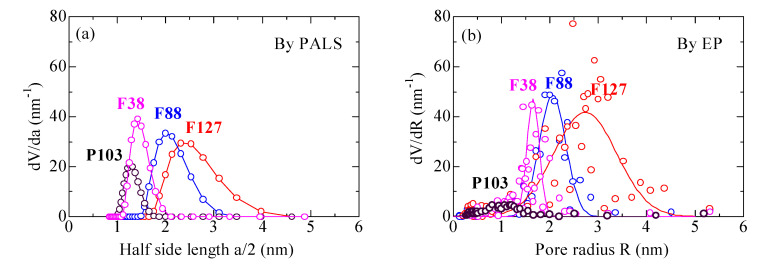
Pore size distributions of the films with various templates calculated by (**a**) PALS and (**b**) EP.

Pore size distributions (PSDs) of the films with various templates are displayed in Figure 4, calculated (a) by PALS according to the RTE model [10,22,23] from the longest-lived o-Ps lifetime distributions of Figure 3 and (b) by EP based on the BJH model [39] from the corresponding desorption isotherms of Figure 2. For the F38 templated film with worm-like channels, the pore size was calculated by PALS under assumption of rectangular pores because of its high interconnectivity, while the pore size of cage-like pores was calculated assuming cubic pores for the other three films. Predominant peaks of PSD can be seen for all films. The average pore sizes deduced by PALS are very close to those by EP. The average pore side lengths by PALS are about 2.60, 2.96, 4.37 and 5.63 nm for the films with P103, F38, F88 and F127, respectively, and the BJH pore diameters are around 2.20, 3.25, 4.18 and 5.46 nm, respectively.

Nevertheless, as demonstrated in Figure 4b, the height of BJH PSD peak for the film with P103 is very low because of the inaccessibility for gas molecules into closed pores. However, positrons can be implanted inside the film and Ps atoms are preferentially localized in closed pores and annihilate therein, showing an obvious PSD peak of P103 templated film in Figure 4a. The full width at half maximum of PSD by PALS is much higher than that by EP for the film templated by F38, because positrons can be trapped by micro/mesopores that smaller than the minimum size pores detected by EP, resulting in a broader PSD. The result indicates that PALS has more advantages in characterizing closed pores and meso/micropore size distributions in mesoporous thin films.

For the films templated by F88 and F127, both the PSD by PALS and the BJH PSD are much broader than those for the film with F38. As is revealed by the hysteresis loops as well as the TEM observations [5], uniform worm-like pores are formed in the film with F38, and more generous cage-like pores exist in the films with F88 and F127. The cage-like pores consist of larger cages and relatively smaller connecting channels. For instance, the mean pore size of the connecting channels between neighboring cages was determined around 2.5 nm for the film templated by F127 in the same way as previously published [2]. Heterogeneity of coexistence of cages and tubular channels probably results in broader PSDs in the films templated by F88 and F127.

### 3.3. Comparison of Open Porosity and Pore Size by PALS and EP

**Figure 5:**
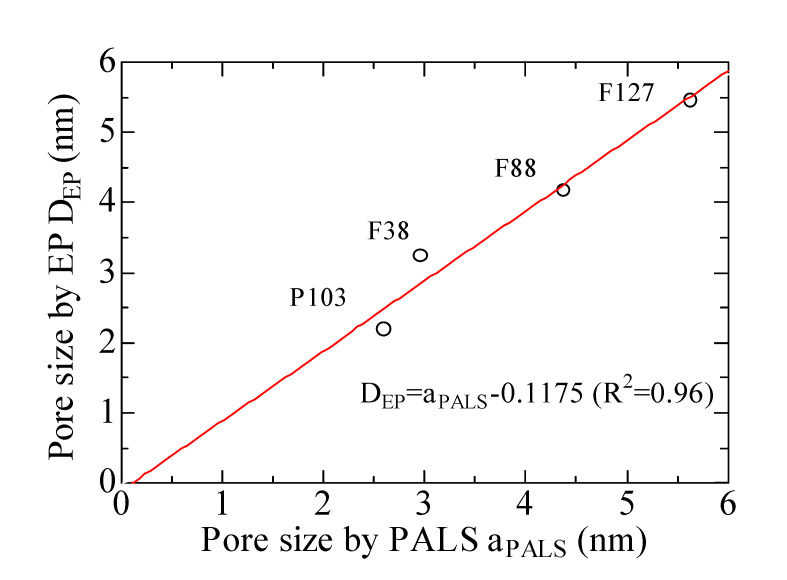
Pore sizes determined by EP of the films with various surfactants versus pore sizes by PALS.

The comparison of the average pore sizes obtained by PALS (*a*_PALS_) and EP (*D*_EP_) is plotted in Figure 5. The circle represents the pore size calculated by the two methods. All the pore sizes fall on a fitted line of slope *k* = 1 with the fitting variation of R^2^ = 0.96, as seen red line in Figure 5. The linear fitting R^2^ approaches to 1, indicating that both PALS and EP can precisely measure the mesopore size of porous silica films prepared with various polymeric templates. Further, the fitting result shows that relatively larger average pore sizes were obtained by PALS, because Ps atoms were preferentially localized in larger pores.

**Figure 6:**
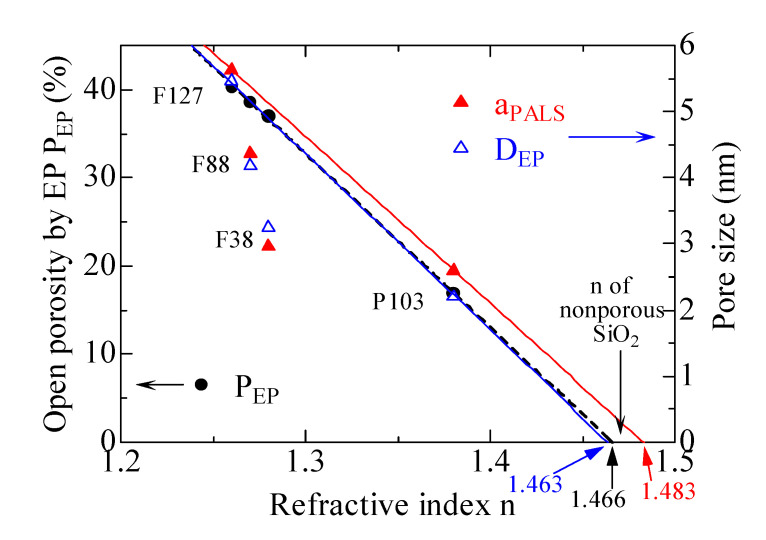
Open porosity measured by EP (black solid circles) and pore size by PALS (red solid triangles) and EP (blue open triangles), respectively, as a function of the refractive index as well as the copolymer templates.

The open porosities measured by EP (*P*_EP_) (black solid circles) and pore sizes obtained by PALS (red solid triangles) and EP (blue open triangles), respectively, versus the refractive index as well as the copolymer templates are exhibited in Figure 6. The black dashed line is the linear fitting results of the refractive index dependence of the open porosity. The *P*_EP_ is evaluated by the simplified Lorentz–Lorenz equation, as formulated in Equation (1) with the measured *n*_o_. A linear relationship between the open porosity and the refractive index appears for the films with various triblock copolymers. Interestingly, this relation offers the refractive index *n* = 1.466 for nonporous silica if the line is extrapolated to open porosity for *P*_EP_ = 0.

The red and blue lines in Figure 6 represent the linear fitting results of the refractive index dependence of “closed” pore sizes by PALS and EP, respectively. Although the mesopores in the film synthesized by F127 are well interconnected and open to the film surface, the diffusion length of Ps atoms in it is relatively short, so from the point of view of Ps diffusion, the pores in both F127 and P103 templated films are so-called “closed”. For the films with “closed” pores, it seems that the refractive index of nonporous SiO_2_ can be obtained for both PALS and EP, when the pore sizes are epitaxial to 0. For the fitted bule line investigated by EP, the intercept on the horizontal axis, which represents the refractive index of nonporous SiO_2_, is 1.463, near to an ideal value of bulk fused quartz (1.460) [40]. Likewise, the red linear correlation between the pore size by PALS and the refractive index gives a refractive index of nonporous SiO_2_ of 1.483, comparable to that from EP. The results indicate that the open porosities/interconnectivities and pore sizes measured by PALS are in good agreement with those by EP.

## 4. Conclusions

PAES and PALS based on slow positron beams as well as EP combined with heptane adsorption were applied to investigate the open mesoporosity/interconnectivity and mesopore size for the mesoporous silica films templated by different triblock copolymers. Both the o-Ps 3γ annihilation fractions and heptane adsorption–desorption isotherms show more closed pores in the film with P103 and well connected mesopores in the respective films with F38, F88 and F127. The linear relationship between the pore sizes with the slope of 1 estimated by PALS and EP displays a good consistency with each other. For the film fabricated by P103, the PSD peak deduced by the longest-lived o-Ps lifetimes is more remarkable than BJH peak by EP, which signifies higher sensitivity for PALS in determining closed pores than EP. The EP combined with heptane adsorption is a useful method for calibrating the mesopore size in highly interconnected films, while PALS is a novel, complementary probe for both closed and open pores.

## Data Availability

Data contained within the article regarding the *I*_3γ_-*E*_in_ curves and the heptane adsorption–desorption isotherms of the films with F38 and F127 were published previously by American Physical Society and are available at https://doi.org/10.1103/PhysRevB.86.075415 (accessed on 17 May 2021) with the permission of Copyright 2012 American Physical Society. The positron annihilation lifetime spectra of the films with P103, F38 and F127 were reported previously in Elsevier and are available at https://doi.org/10.1016/j.radphyschem.2006.03.036 (accessed on 17 May 2021) with the permission of Copyright 2006 Elsevier.

## Supplementary Materials

The following are available online at https://www.mdpi.com/article/10.3390/ma14123371/s1, Figure S1: The schematic diagram of the apparatus of heptane adsorption.
