# Supplementary figures and images for: Open Porosity and Pore Size Distribution of Mesoporous Silica Films Investigated by Positron Annihilation Lifetime Spectroscopy and Ellipsometric Porosimetry
Source: Materials (Basel). 2021 Jun 18;14(12):3371. doi: 10.3390/ma14123371 (PMC8235386; doi:10.3390/ma14123371)

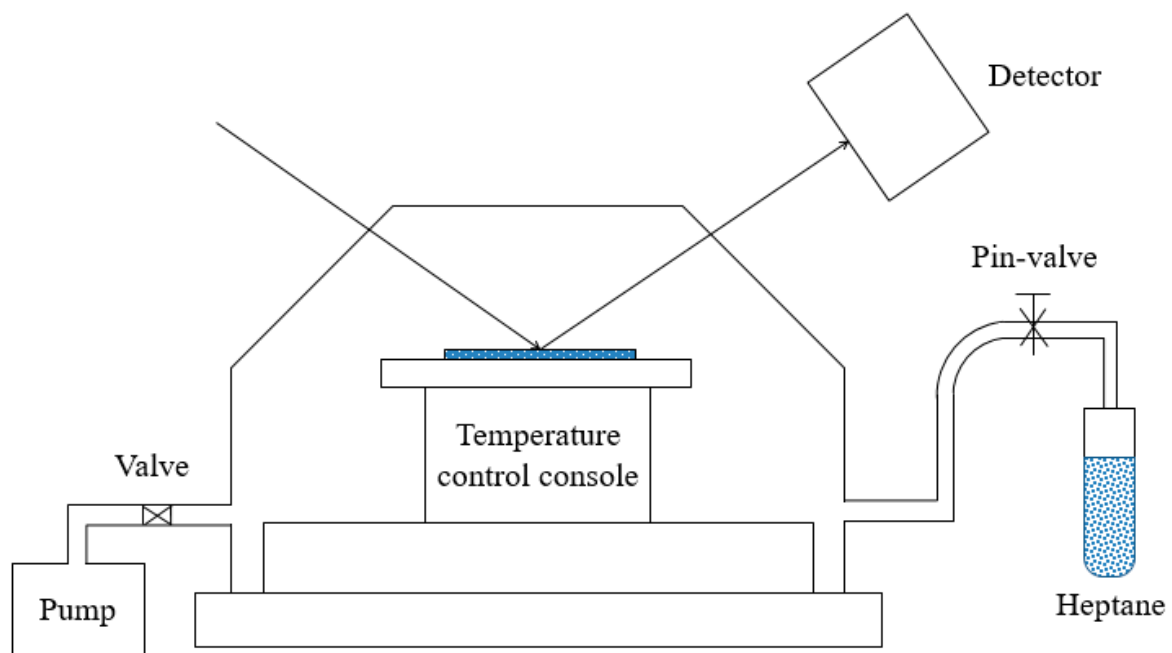

**Figure S1.** The schematic diagram of the apparatus of heptane adsorption.

Supplement: Supplementary file 1 [file materials-14-03371-s001.zip › materials-1244656-supplementary.pdf]
